# Cavity-enhanced superconductivity in MgB_2_ from first-principles quantum electrodynamics (QEDFT)

**DOI:** 10.1073/pnas.2415061121

**Published:** 2024-12-05

**Authors:** I-Te Lu, Dongbin Shin, Mark Kamper Svendsen, Hannes Hübener, Umberto De Giovannini, Simone Latini, Michael Ruggenthaler, Angel Rubio

**Affiliations:** ^a^Theory Department, Max Planck Institute for the Structure and Dynamics of Matter and Center for Free-Electron Laser Science, 22761 Hamburg, Germany; ^b^Department of Physics and Photon Science, Gwangju Institute of Science and Technology, 61005 Gwangju, Republic of Korea; ^c^Novo Nordisk Foundation Quantum Computing Programme, Niels Bohr Institute, University of Copenhagen, 2100 Copenhagen, Denmark; ^d^Dipartimento di Fisica e Chimica-Emilio Segrè, Università degli Studi di Palermo, I-90123 Palermo, Italy; ^e^Department of Physics, Technical University of Denmark, 2800 Kongens Lyngby, Denmark; ^f^Center for Computational Quantum Physics, The Flatiron Institute, New York, NY 10010

**Keywords:** quantum electrodynamical density functional theory, condensed matter physics, superconductivity, electronic structure, cavity quantum electrodynamics

## Abstract

Strong laser pulses can control superconductivity, inducing nonequilibrium transient pairing by leveraging strong-light matter interaction. Here, we demonstrate theoretically that equilibrium ground-state phonon-mediated superconductive pairing can be affected through the vacuum fluctuating electromagnetic field in a cavity. Using the recently developed ab initio quantum electrodynamical density-functional theory approximation, we specifically investigate the phonon-mediated superconductive behavior of MgB_2_ under different cavity setups and find that in the strong light–matter coupling regime its superconducting transition temperature T_c_ can be enhanced at most by ≈10% in an in-plane (or out-of-plane) polarized and realistic cavity via photon vacuum fluctuations. The results highlight that strong light–matter coupling in extended systems can profoundly alter material properties in a nonperturbative way by modifying their electronic structure and phononic dispersion at the same time. Our findings indicate a pathway to the experimental realization of light-controlled superconductivity in solid-state materials at equilibrium via cavity materials engineering.

## Significance

Here, we introduce a method to enhance phonon-mediated superconductivity using photon quantum vacuum fluctuations in an optical cavity. By applying quantum electrodynamical density-functional theory, we show that the superconducting transition temperature of MgB_2_ can be significantly increased through coupling with the cavity’s vacuum electromagnetic field. This study reveals a pathway for manipulating material properties at equilibrium, emphasizing the potential of cavity materials engineering for achieving light-controlled superconductivity and other material properties, with broad implications for materials science and condensed matter physics.

The prospect of light-controlled superconductivity has driven a vast range of recent experimental and theoretical efforts (1–5). While strong lasers facilitate material control in the out-of-equilibrium regime, an alternative approach involves using the quantum fluctuations of electromagnetic fields to modify material properties at equilibrium within cavities: the strong interaction between matter and photon fields in a cavity (6, 7) gives rise to the emerging fields of polaritonic chemistry (8–14) and cavity materials engineering (15, 16) that promise to revolutionize the way we perceive materials science (1, 17). However, while there is convincing experimental evidence supporting the modification of molecules within cavities through vacuum fluctuations (18), equivalent findings for extended materials are scarce (19–22). Research on polaritons primarily considers the combined response of light and matter excitations (23–28), rather than directly investigating material changes.

Most theoretical predictions for cavity-controlled solid-state materials have relied on model Hamiltonians (29–36). Notably, the control of superconductivity in a cavity has been proposed and explored through various approaches (37–42). For instance, coupling photons to phonons modifies the electron–phonon coupling and phonon frequency (37), or generating nonequilibrium states through the quantum Eliashberg effect (39). A few recent experiments support theoretical proposals to alter ground-state material properties via photon fluctuations, for instance, the breakdown of topological protection in the quantum Hall effect in two-dimensional electron gases (19, 22, 43) and the renormalization of the critical temperature for the metal-to-insulator transition in layered TaS_2_ within a Fabry-Pérot cavity (20).

However, developing efficient theoretical methods for complex light–matter coupling in realistic extended materials within a cavity is challenging due to the vast number of degrees of freedom required to describe light and matter on the same footing. QEDFT presents an exact and practical solution, by shifting the complexity in the degrees of freedom into a search for functionals of the electronic density (15, 44). As opposed to standard DFT, where a functional accounting for the electronic exchange and correlation is required, one here needs to approximate the electron–photon interaction. The recently developed QEDFT method (45, 46), which is inherently nonperturbative, can describe strong light–matter interactions in extended systems embedded in arbitrary electromagnetic environments like optical cavities (47).

Here, we demonstrate, using QEDFT, the tuneability of the superconducting transition temperature (T_c_) in MgB_2_, a phonon-mediated superconductor, through coupling to the vacuum fluctuations of an optical cavity. MgB_2_ is a conventional phonon-mediated superconductor with a high T_c_ of ≈39 K (48). Its superconducting behavior is well described by standard DFT methods, which correctly predict its T_c_ and two anisotropic superconducting gaps originating from Boron π and σ bands (49). QEDFT predicts that the T_c_ of MgB_2_ can be enhanced by up to 73% when strongly coupled to electromagnetic fluctuations of the cavity vacuum that are polarized along the materials stacking planes. Rotating the cavity polarization into the direction perpendicular to the planes, instead, can lead to an enhancement of up to 40%. We ascribe this change in the critical temperature to the compounding effect of both the enhanced electron–phonon coupling and the renormalized phonon frequencies of the ground state. QEDFT has gradually matured to the point where it can be applied to the simulation and analysis of light–matter interactions in complex material systems, providing a tool for advancing material manipulation through tailored fluctuating electromagnetic fields. This work shows that cavity materials engineering can achieve profound changes in equilibrium material properties, beyond the perturbative regime.

## Methodology

Light–matter coupled systems in the nonrelativistic regime are described in QED by the PF Hamiltonian (15). The effect of the electromagnetic modes of an optical cavity can be described in terms of a few effective modes whose coupling to matter depends on both material and cavity properties (50). In the velocity gauge within the long-wavelength approximation (50) the PF Hamiltonian reads as follows (in the Hartree atomic units):

[1] $$\begin{array}{c} \begin{array}{cc} {\hat{H}}_{\mathrm{PF}} & =\frac{1}{2}\sum_{l=1}^{N_{e}}{\left(-i\nabla_{l}+\frac{1}{c}\hat{A}\right)}^{2}+\frac{1}{2}\sum_{l\neq k}^{N_{e}}w\left(r_{l},r_{k}\right)\\ & \;+\sum_{l=1}^{N_{e}}v_{\mathrm{ext}}\left(r_{l}\right)+\sum_{\alpha =1}^{M_{p}}\omega_{\alpha }\left({\hat{a}}_{\alpha }^{†}{\hat{a}}_{\alpha }+\frac{1}{2}\right), \end{array} \end{array}$$

where l (α) is the index for electrons (effective photon modes), $N_{e}$ ($M_{p}$) is the number of electrons (effective photon modes), $w(r_{l},r_{k})$ and $v_{\mathrm{ext}}\left(r_{l}\right)$ are the Coulomb interaction among electrons and between electrons and nuclei, respectively, $r_{l}$ is the position for the lth electron, $\omega_{\alpha }$ and ${\hat{a}}_{\alpha }$ (${\hat{a}}_{\alpha }^{†}$) are the frequency and annihilation (creation) operator of the αth effective photon mode, respectively. The vector potential (or photon field) operator in the long-wavelength approximation is $\hat{A}=c\sum_{\alpha =1}^{M_{p}}\lambda_{\alpha }\varepsilon_{\alpha }\left({\hat{a}}_{\alpha }^{†}+{\hat{a}}_{\alpha }\right)/\sqrt{2\omega_{\alpha }}$, where c is the speed of light and $\varepsilon_{\alpha }$ the polarization of the αth effective photon mode with the effective mode strength $\lambda_{\alpha }=\sqrt{4\pi /\Omega_{\alpha }}$ (the effective mode volume $\Omega_{\alpha }$). Using the electron–photon exchange approximation (45, 46), we reduce the degrees of freedom of the PF Hamiltonian by recasting the problem into a purely electronic one. We do so by mapping the electromagnetic vacuum fluctuations ($\Delta {\hat{A}}_{\alpha }$) to fluctuations of the electronic paramagnetic current ($\Delta {\hat{J}}_{p}$) of the material, i.e., $\Delta {\hat{A}}_{\alpha }\propto \varepsilon_{\alpha }\cdot \Delta {\hat{J}}_{p}$ (46). Within QEDFT (15, 44), we can then apply the KS scheme to formulate the problem with a purely electronic Hamiltonian

$${\hat{H}}_{\mathrm{KS}}=-\frac{1}{2}\nabla^{2}+v_{\mathrm{ext}}\left(r\right)+v_{\mathrm{Hxc}}\left(r\right)+v_{\mathrm{pxc}}\left(r\right),$$

where $v_{\mathrm{ext}}\left(r\right)$ is the external potential from the nuclei, $v_{\mathrm{Hxc}}\left(r\right)$ the Hartree and exchange-correlation (xc) potential from electron–electron interaction, and $v_{\mathrm{pxc}}\left(r\right)$ the electron–photon exchange-correlation potential. The Coulomb xc-potential can be obtained using commonly used DFT functionals (51). In this work, the electron–photon exchange-correlation potential, instead, is approximated as the electron–photon exchange potential within the LDA (45, 46) as the solution of the following Poisson equation

[2] $$\nabla^{2}v_{\mathrm{pxLDA}}\left(r\right)=-\sum_{\alpha =1}^{M_{p}}\frac{2\pi^{2}{\tilde{\lambda }}_{\alpha }^{2}}{{\tilde{\omega }}_{\alpha }^{2}}{\left({\tilde{\varepsilon }}_{\alpha }\cdot \nabla \right)}^{2}{\left(\frac{3\rho (r)}{8\pi }\right)}^{2/3},$$

where ρ(r) is the electron density and the tilde (^~^) indicates renormalized quantities (*Mapping Photonic Fluctuations to Electronic Currents*). Within this method, the different cavity configurations are obtained by varying the ratio of the mode strength $\lambda_{\alpha }$ and the effective cavity photon frequency $\omega_{\alpha }$, as this ratio ($\lambda_{\alpha }/\omega_{\alpha }$), together with photon polarization, is the only parameter that affects the electron–photon exchange potential in our simulations. Here, instead of providing the details of a realistic cavity setup, we use these two variables, $\lambda_{\alpha }$ and $\omega_{\alpha }$, to encode the detailed information of the cavity setup such as the mode volume, photon frequency, cavity material, and so on. We treat these two variables as free parameters. The ratio of these two variables ($\lambda_{\alpha }/\omega_{\alpha }$) can be reached up to 0.1 in phonon- and plasmon-polariton based cavities (52, 53). We also include the electron–photon exchange potential in solving cavity-modified phonon dispersions with the density functional perturbation theory (*Computing Cavity-Modified Phonon Dispersion and Superconductivity*). All the computational details can be found in *Computational Details* and a comprehensive description of the derivation of the electron–photon exchange functional used here can be found in refs. 45 and 46.

## Results

### Cavity Modification of the Superconducting Critical Temperature.

**Fig. 1.**
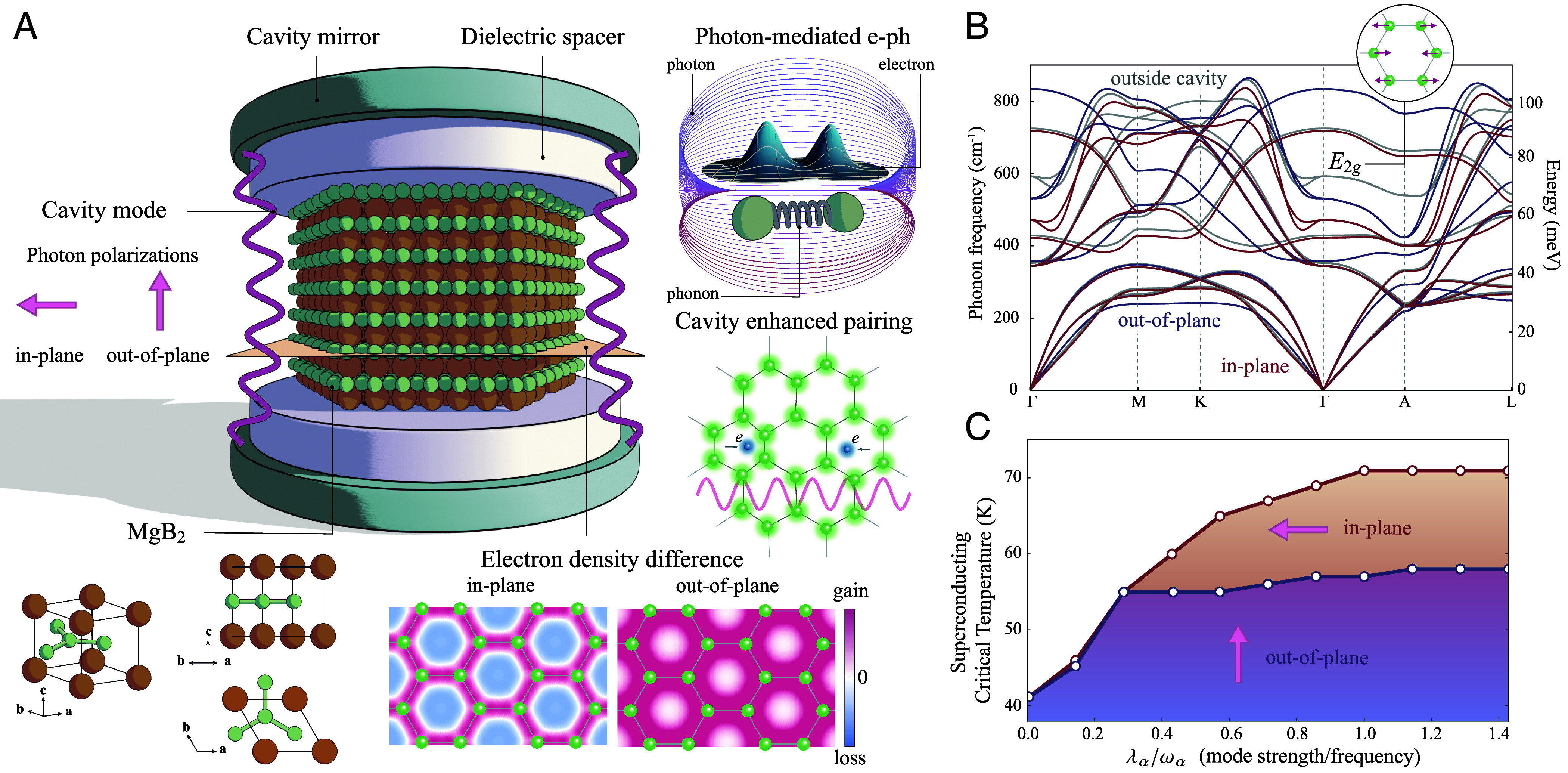
Cavity-modified superconductivity. (*A*) MgB_2_ (Magnesium atoms in orange and Boron atoms in green), a phonon-mediated superconductor, is put inside an optical cavity. The detailed information of the cavity and the light–matter interaction between the cavity and MgB_2_ is encoded into the effective mode strength $\lambda_{\alpha }$, frequency $\omega_{\alpha }$, and polarization $\varepsilon_{\alpha }$ of the effective photon modes that MgB_2_ is strongly coupled to. Here, we use two cavity setups: an in-plane polarized cavity with two effective photon modes parallel to the Boron planes and an out-of-polarized cavity with one effective photon mode perpendicular to the Boron planes. We simulate the electronic ground state using QEDFT and the phonon dispersion using the density functional perturbation theory including the light–matter interaction. The electron–phonon coupling is modified (and enhanced) due to the changes of the electronic states and phonons inside the cavity, leading to the enhanced superconductivity. Cavity-induced change in electron density Δρ(r) on the Boron plane in MgB_2_ with the pristine lattice constant modifies the phonon-Raman-mode $E_{2g}$, which mainly drives the superconductivity. (*B*) Ab initio calculated phonon frequency $\omega_{\nu q}$ for the νth phonon branch at the crystal momentum q of MgB_2_ (with the fixed pristine lattice constant) outside and inside the in-plane and the out-of-plane polarized cavity. The $E_{2g}$ Raman mode softens due to the screening of the enhanced electron density within the Boron–Boron σ regime inside a cavity, diminishing the repulsion between Boron atoms. The ratio of the mode strength and photon frequency ($\lambda_{\alpha }/\omega_{\alpha }$) is 1.0 for both cavity setups. (*C*) Superconducting transition temperature as a function of the bare mode strength $\lambda_{\alpha }$ and photon frequency $\omega_{\alpha }$ ratio with the pristine lattice constant. The enhanced superconducting transition temperature is mainly due to the softened $E_{2g}$ mode, which mainly drives the superconductivity of MgB_2_. We estimate that $\lambda_{\alpha }/\omega_{\alpha }$ can reach up to around 0.1 for a polaritonic cavity setup.

The superconductive behavior of MgB_2_ is modified when it is placed inside an optical cavity, illustrated in Fig. 1*A*, which is a simple optical resonator with a planar geometry. The T_c_ of MgB_2_ outside a cavity is well described by Eliashberg theory using the first-principles methods (49, 54), which are constructed from the electronic and phononic structure. Hence, cavity-renormalization of T_c_ can be predicted by using QEDFT to calculate these quantities when dressed by the vacuum fluctuations of light. We analyze two different configurations for the MgB_2_ within the optical cavity. These include 1) an *out-of-plane* configuration, with a single effective photon mode polarized perpendicular to the Boron plane, and 2) an *in-plane* configuration, with two effective photon modes—one polarized along x and the other along y, with y pointing in the Boron–Boron σ bond direction.

We find that the coupling between the cavity and MgB_2_ dresses electrons and thereby renormalizes the forces exerted by the electrons on the nuclei. This is a clear demonstration of the nonperturbative nature of the coupling with consequences on both the electronic and phononic subsystems of the material. While these nonperturbative effects due to the cavity can in principle change the lattice unit cell of MgB_2_, we here consider the DFT-relaxed cell outside the cavity. The dressing of electrons with the photon modes changes the electron density on the Boron plane, concentrating electrons around Boron σ bonds (Fig. 1*A*). In both cavity configurations, this accumulation screens the Coulomb repulsion between Boron ions, leading to a softening of the $E_{2g}$ Raman phonon frequency (Fig. 1*B*). The $E_{2g}$ mode is the phonon mode that primarily drives superconductivity in MgB_2_ (55), and therefore the cavity-induced softening of this mode enhances the T_c_ up to 71 K (in-plane) and 58 K (out-of-plane) in the respective polarized cavities (Fig. 1*C*). We note that in practice, however, the increase in T_c_ is limited by the maximum achievable value of $\lambda_{\alpha }/\omega_{\alpha }$, which is directly related to the strength of the vacuum field fluctuations in the cavity (47). While the paradigmatic, parallel mirror Fabry-Pérot cavity is unlikely to show vacuum field enhancements strong enough to drive significant changes in the T_c_ (50), coupling strengths of at least $\lambda_{\alpha }/\omega_{\alpha }∼0.1$ should be achievable in phonon- and plasmon-polariton based cavity setups (52, 53). We therefore expect that a change in the T_c_ of around 5 K is experimentally reachable with current cavity setups.

**Fig. 2.**
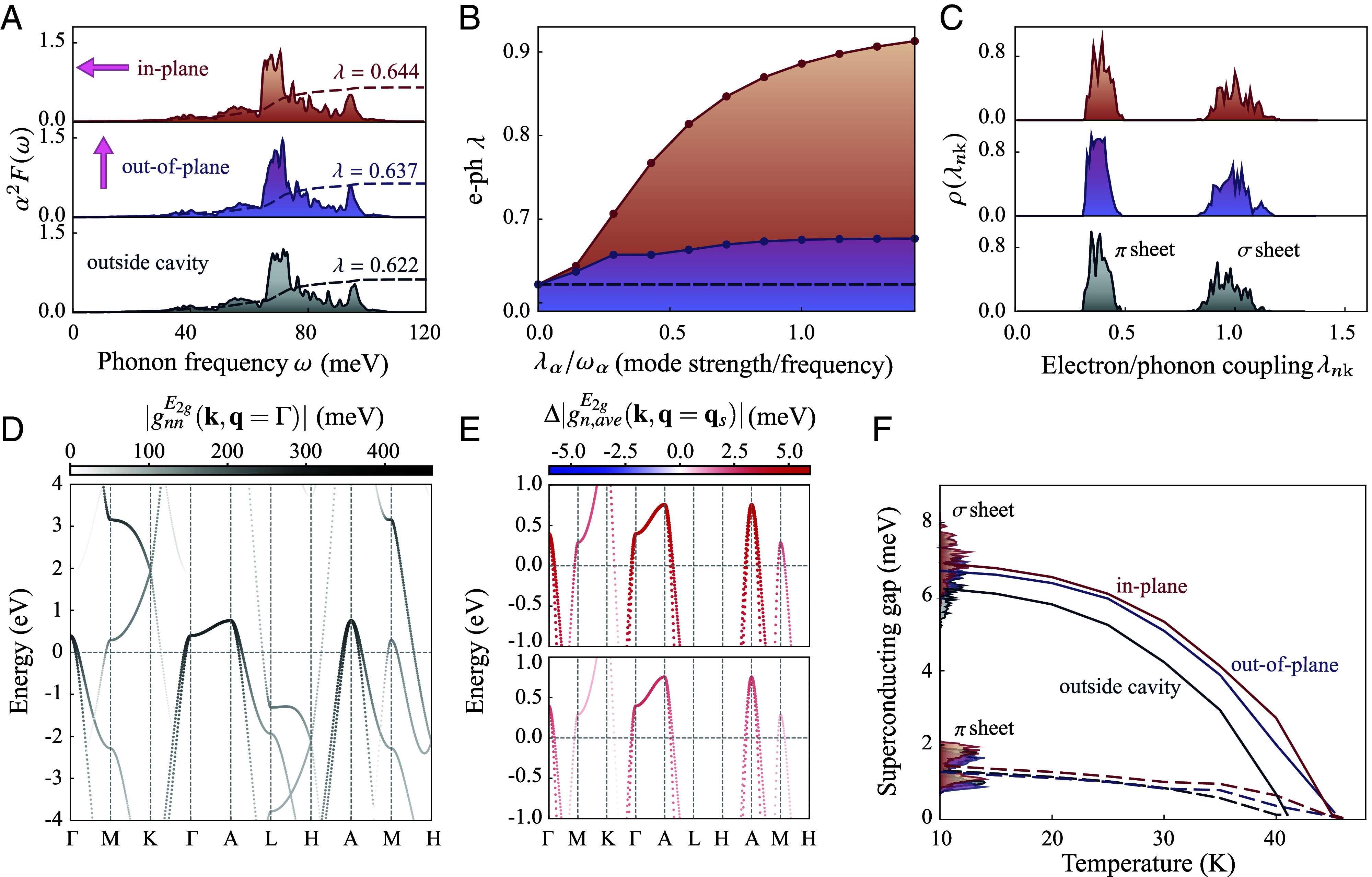
Superconducting quantities in the cavity. (*A*) Isotropic Eliashberg function $\alpha^{2}F\left(\omega \right)$ and total electron–phonon coupling λ (dashed lines) inside and outside the cavity. The main peak intensity corresponding to the $E_{2g}$ phonon mode (around 70 meV) increases inside the cavity, while its frequency slightly shifts down due to the softened $E_{2g}$ mode. (*B*) Total electron–phonon (e-ph) coupling λ as a function of the ratio of the bare mode strength $\lambda_{\alpha }$ and photon frequency $\omega_{\alpha }$; the dashed line is the total electron–phonon coupling outside the cavity. Given a fixed photon frequency, the electron–phonon coupling increases as the mode strength (or light–matter coupling) and saturates at large mode strengths. (*C*) Distribution of the electron–phonon coupling strengths, $\rho (\lambda_{nk})$, for electronic states |nk⟩ (with the band index n and crystal momentum k) at the Fermi energy inside and outside the cavity; lower values correspond to the π sheet, and higher values to the σ sheet. The cavity mainly modifies the electron–phonon couplings of the σ sheet. (*D*) Diagonal electron–phonon matrix elements $g_{\mathrm{nn}}^{E_{2g}}\left(k,q=\Gamma \right)$ (defined in the main text) for the Γ-point phonon $E_{2g}$ mode outside the cavity. The states around the Fermi surface are strongly coupled to the $E_{2g}$ mode. (*E*) Cavity-induced changes in averaged electron–phonon matrix elements (defined in the main text) due to the $E_{2g}$ mode at the nesting momentum $q_{s}$, which connects the electron states across the σ sheet. The electron–phonon coupling matrix elements enhance in both cavity setups. (*F*) Two superconducting gaps—where the higher values correspond to the σ sheet, while the lower ones to the π sheet—as a function of temperature inside and outside the cavity. The solid lines are the averaged gap values for the σ sheet, while the dashed lines are for the π sheet; we also show the histogram of the superconducting gaps at 10 K. T_c_ is determined when the superconducting gaps vanish. All the calculations use the pristine lattice constant for MgB_2_. The ratio of the $\lambda_{\alpha }$ and $\omega_{\alpha }$ used in panels *A* and *C*–*F* is chosen as ≈0.14.

The T_c_ in phonon-mediated superconductors can be determined by solving the mass renormalization function and the superconducting gap using the anisotropic Migdal–Eliashberg theory, which involves the anisotropic electron–phonon coupling matrix, electronic density of states at the Fermi energy, phonon dispersion, and electronic band structure of the material (*Computing Cavity-Modified Phonon Dispersion and Superconductivity*). The Eliashberg spectral function quantitatively describes the probability of an electron emitting or absorbing a phonon at a specific frequency $\omega_{\mathrm{ph}}$. Fig. 2*A* shows that the isotropic Eliashberg function $\alpha^{2}F\left(\omega_{\mathrm{ph}}\right)$ for MgB_2_ is indeed changed inside the cavity. For example, the intensity of the dominant peak at around 70 meV corresponding to the $E_{2g}$ phonon mode increases, compared to the case of the material being outside the cavity. To estimate the changes in T_c_ we then calculate the total electron–phonon coupling strength λ from the isotropic Eliashberg function (*Computing Cavity-Modified Phonon Dispersion and Superconductivity*). Increased λ corresponds to higher superconducting temperatures (56). Consistently, in Fig. 2*B*, λ rises with the photon mode strength $\lambda_{\alpha }$ in both in-plane and out-of-plane polarized cavity, explaining the enhanced T_c_ as shown in Fig. 1*C*.

### Nonperturbative Changes in the Electronic and Phononic Structure.

The shift in the total electron–phonon coupling strength λ may stem from changes in phonon frequency $\omega_{\nu q}$ and electron–phonon matrix elements $g_{mn,\nu }\left(k,q\right)=\left\langle mk+q\right|\partial_{q\nu }V\left|nk\right\rangle$. These matrix elements describe the scattering between the single particle electronic states |nk⟩ (with the band index n and crystal momentum k) and |mk+q⟩ via the ion-electron potential change, $\partial_{q\nu }V$, induced by the νth phonon branch at momentum q. Note that we also include the electron–photon interaction contribution in the ion-electron potential (*Computing Cavity-Modified Phonon Dispersion and Superconductivity*). Examining the electron–phonon matrix elements without light–matter interaction, we find that electronic states at the Fermi surface are strongly coupled to the zone-centered $E_{2g}$ mode due to the large diagonal electron–phonon matrix elements (Fig. 2*D*) and in agreement with existing literature (57). Specifically, to assess the photon-induced changes of the electronic states across the σ sheet of the Fermi surface connected via the phonon momentum $q_{s}$, we compute the average electron–phonon matrix elements between the $E_{2g}$ mode at $q_{s}$ and the three highest valence bands at the zone center Γ, defined as $|g_{n,\mathrm{ave}}\left(\Gamma ,q_{s}\right)|=\sqrt{\sum_{m}^{N_{b}}{\left|g_{nm,\nu }\left(\Gamma ,q_{s}\right)\right|}^{2}/N_{b}}$ (m runs over the three electronic bands and $N_{b}=3$). We find enhanced electron–phonon coupling in both the in-plane and out-of-plane polarized cavity configurations (Fig. 2*E*). This indicates that, in addition to the $E_{2g}$ mode softening, the change in electron–phonon matrix elements is contributing to the enhancement of T_c_.

The superconductivity of MgB_2_ is characterized by two superconducting gaps associated with the pairing of electrons within two different Fermi sheets of π and σ character, respectively. A way to discriminate the influence of the cavity on these two sheets is to analyze the distribution of values of the band- and wave-vector-dependent electron–phonon coupling strength at the Fermi level (*Computing Cavity-Modified Phonon Dispersion and Superconductivity*), shown in Fig. 2*C*. Lower values of electron–phonon coupling strength cluster on the π sheet, and higher values on the σ sheet (49). These two sheets are affected differently by the cavity: the σ sheet is affected most, with a clear shift toward higher values, while the π sheet remains largely unaffected. Both in-plane and out-of-plane polarized cavity increases these values. The consequence of this modification is an asymmetric change of the superconducting gaps, c.f., Fig. 2*F* showing the superconducting gaps of MgB_2_ inside and outside the cavity. The superconducting transition temperature T_c_ can be directly determined from this representation by evaluating the vanishing points of the superconducting gaps as a function of temperature. The calculations yield a T_c_ of 41 K for MgB_2_ outside the cavity, which closely aligns with experimental data (39 K). Note that we use the DFT-relaxed lattice constant, which leads to lower theoretical T_c_ compared to existing literature (around 50 K using the experimental lattice constant) where this discrepancy can be rectified by considering anharmonicity (58) or nonadiabatic phonon dispersion effects (59). The calculations using the experimental lattice constant show a similar trend as described above. The enhanced T_c_ primarily results from the enhanced electron–phonon coupling and the softened $E_{2g}$ phonon mode, rather than changes in the electronic states due to the cavity. To clarify this, we performed an additional calculation using the in-plane-polarized-cavity-modified electronic states under the same light–matter coupling conditions (as in Fig. 2*F*), but with the interatomic force constants from the outside-cavity case. This resulted in a T_c_ of 41 K, which is nearly identical to the outside-cavity scenario.

**Fig. 3.**
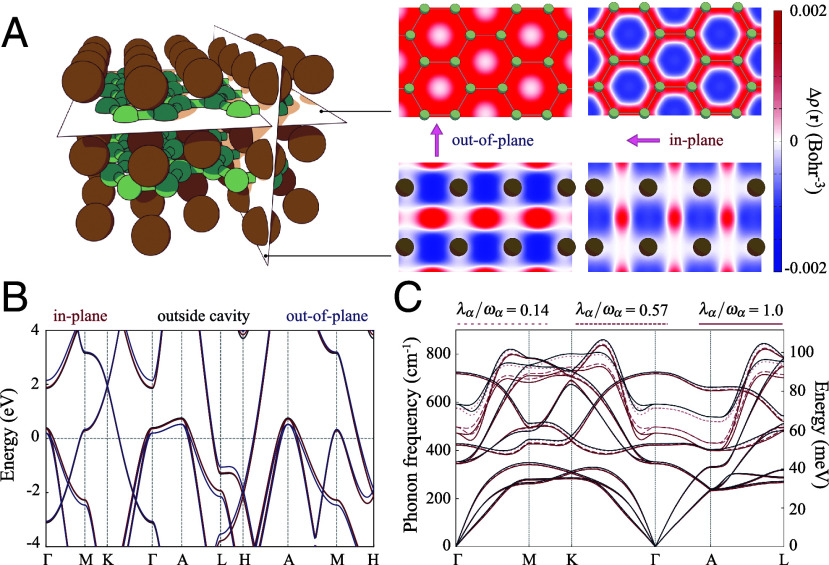
Cavity manipulation of electronic and phononic structure of MgB_2_. (*A*) Cross-sections of the cavity-induced change in electron density Δρ(r) with the out-of-plane and in-plane polarization. (*B*) Cavity-modified electronic band structure for the ratio of the mode strength and photon frequency, $\lambda_{\alpha }/\omega_{\alpha }=1.0$. The general shape of the band structure of MgB_2_ inside the cavity is similar to that outside the cavity. (*C*) Cavity-modified phonon dispersion along the high symmetric lines in the in-plane polarized cavity for three light–matter coupling strengths, $\lambda_{\alpha }/\omega_{\alpha }=0.14$, 0.57, and 1.0. The in-plane polarized cavity mainly modifies and softens the $E_{2g}$ Raman phonon mode.

Now that we have discussed the cavity effect on the phonons and the electron–phonon coupling, we turn to a more detailed analysis of the underlying renormalization of the electronic and phononic structure of the material. Fig. 3*A* shows the cavity-induced changes of the real space electron density in the cavity. In the out-of-plane polarized cavity, the electronic density shifts from the plane of Magnesium atoms to the bond regime between Boron atoms in the Boron plane. Instead, in the in-plane polarized cavity, electrons relocate from the center of the hexagons on the Boron plane to the σ bonds on the same plane. This behavior can be understood through a simple physical explanation. The Hamiltonian within the electron–photon exchange approximation contains the current–current correlation term ${\left({\tilde{\varepsilon }}_{\alpha }\cdot {\hat{J}}_{p}\right)}^{2}$ (46), which counters the kinetic energy aligned with photon field polarization. This equivalently increases the electron’s *physical mass* in the polarization direction (60), because electrons interact strongly with the virtual photons inside the cavity. Therefore, in the in-plane polarized cavity, the enhanced electron physics mass confines electrons closer to the potential energy local minima on the Boron planes, resulting in more electron density in the bond regimes, compared to the center regime of the hexagons.

These changes in the electron density directly impact the electron and phonon dispersion within the cavity. Fig. 3*B* compares electronic band structures near the Fermi energy of MgB_2_ outside and within a cavity. While the general shape of the inside-cavity band structure (and Fermi surface and density of states) resembles that outside the cavity, the light–matter interaction induces modifications that are nonuniform throughout the Brillouin zone, with energy shifts in the meV range. Fig. 3*C* shows the modification of the phonon dispersion within the in-plane polarized cavity with different light–matter couplings ($\lambda_{\alpha }/\omega_{\alpha }$) from 0.14 to 1.0. The light–matter interaction mainly alters the $E_{2g}$ mode in the in-plane polarized cavities. The large change in the phonon frequency along Γ-A is consistent with the strong electron–phonon coupling along that path (57), while acoustic and low-energy optical phonons (below 50 meV) are less affected, because of their weak electron–phonon coupling. Another way of understanding the changes in the phonon frequency is to consider the modification of the dressed potential energy surface (polaritonic surface) induced by the light–matter interaction (61). Notably, the cavity affects various phonon modes across a broad crystal momentum range and not only around the zone center, which is within the light-cone, i.e., within the momentum range of the photons.

## Discussion and Conclusion

On general grounds, this work puts forward a mechanism of light-enhanced superconductivity in the ground state of a material, without exciting the matter with classical fields. This constitutes a paradigm shift, where material phases can be modified and explored as a function of light–matter coupling strength in addition to standard parameters like temperature or pressure, which has been used before to change the superconducting temperature of MgB_2_ (62). The intuitive physical picture that describes how vacuum photon fluctuations affect electrons and phonons, and consequently phonon-mediated superconductivity within a cavity, is as follows: electrons in the crystal, bound by the potential landscape created by the ions, experience enhanced localization due to the interaction with virtual photons from the quantum vacuum. This promotes localization around potential local minima, such as the Boron–Boron σ bonds in MgB_2_. The electron accumulation along these bonds screens Coulomb repulsion between Boron ions, leading to decreased interatomic force constants and softening of the $E_{2g}$ mode, which happens to be the main phonon mode for superconductivity in MgB_2_. Within our developed QEDFT scheme (45, 46) and cavity setup, the predicted superconducting transition temperature T_c_ here depends solely on the ratio of the effective mode strength $\lambda_{\alpha }$ (50) and the photon frequency $\omega_{\alpha }$. High light–matter coupling strength ($\lambda_{\alpha }/\omega_{\alpha }$) can be achieved by reducing the effective photonic mode frequency or by decreasing the effective mode volume ($\lambda_{\alpha }∼1/\sqrt{\Omega_{\alpha }}$). The attainment of coupling strengths necessary to observe enhanced T_c_ depends on the details of an experimental setup, such as the mode volume, frequency, polarization of the effective photon modes, the thickness of the sample, and the quality of the cavity, and so on.

However, to observe enhanced T_c_ in MgB_2_ within an in-plane-polarized cavity with a fixed photon frequency (e.g., 150 meV), we recommend using a thin MgB_2_ sample, as the effective coupling strength diminishes with thickness due to the increased plasma frequency (60). Based on the macroscopic dielectric function of bulk MgB_2_ calculated within the RPA (*Computational Details*), the skin depth of the photon field in MgB_2_ in the THz frequency region is approximately 25 nm, consistent with experimental estimates (63). While the skin depth measures field propagation in the material, it does not account for Fresnel reflection at the dielectric–metal interface, which further limits cavity field penetration. This surface reflection can be mitigated by tuning the refractive index of the dielectric spacer (Fig. 1*A*) to reduce the impedance mismatch between the cavity and the MgB_2_ sample. Therefore, the sample thickness should not exceed 25 nm, and, in general, the dielectric spacer material must be carefully selected as it has a direct impact on the light–matter coupling in the cavity (27, 31). We highlight that the above-proposed mechanism of cavity-enhanced superconductivity is a general one. Still, we anticipate it to potentially lead to a reduction in T_c_ for other materials inside a cavity.

Last, we would like to mention that in our QEDFT simulations, the light–matter coupling strength is treated as a free parameter but can be computed using macroscopic quantum electrodynamics (47), which considers the effects of the cavity material on photon fields via the dyadic Green’s function. Similarly, another work (42) separates the longitudinal and vectorial components of the photon fields, showing that the longitudinal component, which is not considered here, plays an important role in subwavelength cavities, such as plasmonic-based cavities.

In conclusion, we have demonstrated that modifying the ground state of a phonon-mediated superconductor strongly coupled to a cavity can enhance its superconducting transition temperature. By using the advanced QEDFT method on MgB_2_, we have illustrated how the interaction between light and matter inside the cavity alters the electron density of the ground state. This results in nonperturbative modifications of the electronic band structure and the $E_{2g}$ phonon mode of MgB_2_, which is the phonon directly contributing to the change in the superconducting transition temperature. The nonperturbative nature of the mechanism shows that cavity materials engineering should be pursued within first-principles approaches.

## Materials and Methods

### Mapping Photonic Fluctuations to Electronic Currents.

Starting with the nonrelativistic PF Hamiltonian shown in the main text, Eq. **1**, the quadratic (or diamagnetic) term ${\hat{A}}^{2}$ can be directly incorporated into the bare frequency $\omega_{\alpha }$ (64), thereby simplifying the equations. This transformation converts the bare photons into dressed photons, characterized by the dressed photon frequency ${\tilde{\omega }}_{\alpha }$. In the case of multiple modes coupled to the matter the diamagnetic is off-diagonal, i.e., coupling different modes. Hence, the dressed photon frequency has to be obtained by solving the eigenvalues of the following real and symmetric matrix, $W_{\alpha \alpha^{\prime }}=\omega_{\alpha }^{2}\delta_{\alpha \alpha^{\prime }}+N_{e}\lambda_{\alpha }\lambda_{\alpha^{\prime }}\varepsilon_{\alpha }\cdot \varepsilon_{\alpha^{\prime }}$ (45, 46), which can be diagonalized using an orthonormal matrix U, such that $Q=UWU^{T}$ with eigenvalues ${\tilde{\omega }}_{\alpha }^{2}$, for example, the dressed photon frequency for one photon mode is ${\tilde{\omega }}_{\alpha }=\sqrt{\omega_{\alpha }^{2}+N_{e}\lambda_{\alpha }^{2}}$. The mode strength ${\tilde{\lambda }}_{\alpha }$ and polarization direction ${\tilde{\varepsilon }}_{\alpha }$ of the dressed photon mode α can be obtained via the transformation (45) ${\tilde{\lambda }}_{\alpha }{\tilde{\varepsilon }}_{\alpha }=\sum_{\beta =1}^{M_{p}}U_{\alpha \beta }\lambda_{\beta }\varepsilon_{\beta }$, where $\varepsilon_{\alpha }$ (${\tilde{\varepsilon }}_{\alpha }$) is normalized. The PF Hamiltonian with the dressed photon modes becomes

$$\begin{array}{cc} {\hat{\tilde{H}}}_{\mathrm{PF}} & =-\frac{1}{2}\sum_{l=1}^{N_{e}}\nabla_{l}^{2}+\frac{1}{2}\sum_{l\neq k}^{N_{e}}w\left(r_{l},r_{k}\right)+\sum_{l=1}^{N_{e}}v_{\mathrm{ext}}\left(r_{l}\right)\\ & \;+\frac{1}{c}\hat{\tilde{A}}\cdot {\hat{J}}_{p}+\sum_{\alpha =1}^{M_{p}}{\tilde{\omega }}_{\alpha }\left({\hat{\tilde{a}}}_{\alpha }^{†}{\hat{\tilde{a}}}_{\alpha }+\frac{1}{2}\right), \end{array}$$

with the paramagnetic current ${\hat{J}}_{p}=\sum_{l=1}^{N_{e}}\left(-i\nabla_{l}\right)$, the annihilation (creation) operator ${\hat{\tilde{a}}}_{\alpha }$ (${\hat{\tilde{a}}}_{\alpha }^{†}$) for the dressed photon mode α, and with these new operators the vector potential read $\hat{A}=c\sum_{\alpha =1}{\tilde{\lambda }}_{\alpha }{\tilde{\varepsilon }}_{\alpha }\left({\hat{\tilde{a}}}_{\alpha }^{†}+{\hat{\tilde{a}}}_{\alpha }\right)/\sqrt{2{\tilde{\omega }}_{\alpha }}$.

To simulate the effect of the quantum fluctuation of the photon modes on the electronic subsystem, ref. 46 suggests replacing the photon fluctuations with the electron paramagnetic current fluctuations in the PF Hamiltonian. Substituting the vector potential operator $\hat{A}$ with the matter paramagnetic current operator ${\hat{J}}_{p}$ in the PF Hamiltonian yields another Hamiltonian ${\hat{H}}_{B}$ (see below) capable of capturing photon field fluctuations through current–current fluctuations. This allows mapping the quantum fluctuations of photon modes to fluctuations of the electronic current, represented as ${\left({\tilde{\varepsilon }}_{\alpha }\cdot {\hat{J}}_{p}\right)}^{2}$. The static version of the Hamiltonian ${\hat{H}}_{B}$ reads

$$\begin{array}{cc} {\hat{H}}_{B} & =-\frac{1}{2}\sum_{l=1}^{N_{e}}\nabla_{l}^{2}+\frac{1}{2}\sum_{l\neq k}^{N_{e}}w\left(r_{l},r_{k}\right)+\sum_{l=1}^{N_{e}}v_{\mathrm{ext}}\left(r_{l}\right)\\ & \;+\sum_{\alpha =1}^{M_{p}}\frac{{\tilde{\omega }}_{\alpha }}{2}-\sum_{\alpha =1}^{M_{p}}\frac{{\tilde{\lambda }}_{\alpha }^{2}}{2{\tilde{\omega }}_{\alpha }^{2}}{\left({\tilde{\varepsilon }}_{\alpha }\cdot {\hat{J}}_{p}\right)}^{2}, \end{array}$$

where the final term, a current–current correlation operator ${\left({\tilde{\varepsilon }}_{\alpha }\cdot {\hat{J}}_{p}\right)}^{2}$, counteracts the kinetic energy operator and enhances the physical mass of the electron along the polarization direction as the mode strength increases. The associated physical picture is that, in the strong coupling regime, electrons exhibit increased effective mass along the polarization direction, resembling a more *classical* behavior, as they tend to accumulate in regions of minimum external potential (45, 60).

Similar to standard DFT calculations (51), we can use an auxiliary noninteracting system that incorporates the interaction between light and matter, known as the KS system designed to replicate the electron density of the material coupled to cavity photons. The KS Hamiltonian incorporating the light–matter interaction can be expressed as follows (45, 46):

$${\hat{H}}_{\mathrm{KS}}=-\frac{1}{2}\nabla^{2}+v_{\mathrm{KS}}\left(r\right)=-\frac{1}{2}\nabla^{2}+v_{\mathrm{ext}}\left(r\right)+v_{\mathrm{Hxc}}\left(r\right)+v_{\mathrm{pxc}}\left(r\right),$$

where the KS potential $v_{\mathrm{KS}}\left(r\right)$ consists of the external potential from the nuclei $v_{\mathrm{ext}}\left(r\right)$, the Hartree and (longitudinal) exchange-correlation (xc) potential from electron–electron interaction $v_{\mathrm{Hxc}}\left(r\right)$, and the electron–photon (transverse) exchange-correlation potential $v_{\mathrm{pxc}}\left(r\right)$. The Coulomb (longitudinal) xc potential can be obtained using commonly used DFT functionals like the local density approximation (LDA) or PBE (51). On the other hand, the electron–photon (transverse) exchange-correlation potential is approximated using the electron–photon exchange potential within the LDA (45, 46), c.f., Eq. **2** in the main text. To describe the interactions between the ions and the valence electrons, we use the pseudopotential method to separate the core and valence electrons to reduce the computational cost (51) and then focus only on the valence electron density. Once we solve the KS Hamiltonian in a self-consistent way—that is, the Hamiltonian depends on the electron density, and the density depends on the solution of the Hamiltonian—we get the eigenvalue $\epsilon_{nk}$ and eigenstates $|\psi_{nk}\rangle$ for the electronic state |nk⟩ with band index n and crystal momentum k in the Brillouin zone, i.e., ${\hat{H}}_{\mathrm{KS}}\left(k\right)|\psi_{nk}\rangle =\epsilon_{nk}\left|\psi_{nk}\right\rangle$.

### Computing Cavity-Modified Phonon Dispersion and Superconductivity.

Once we have the ground state of the material coupled to cavity photons, we can examine its phonon properties using DFPT (65). To incorporate the light–matter interaction, we solve the following linear system iteratively to get the induced change of the wave function $\partial \psi_{i}\left(r\right)/\partial \mu$ and the perturbation potential due to the atomic displacement $\partial v_{\mathrm{KS}}\left(r\right)/\partial \mu$ (or $\partial_{q\nu }V$ for each photon mode shown in the main text):

$$\left[-\frac{1}{2}\nabla^{2}+v_{\mathrm{KS}}\left(r\right)-\varepsilon_{i}\right]P_{c}\frac{\partial \psi_{i}\left(r\right)}{\partial \mu }=-P_{c}\frac{\partial v_{\mathrm{KS}}\left(r\right)}{\partial \mu }\psi_{i}\left(r\right),$$

where $P_{c}$ is the projector on the conduction bands, and it can be expressed as $P_{c}=1-P_{v}$, where $P_{v}=\sum_{i}|\psi_{i}\rangle \langle \psi_{i}|$ is the projector on the valence bands. The variable μ is a shorthand notation of the atomic displacement $u_{\nu s\beta }$, where ν is the index for the Bravais lattice vectors, s the index for the atoms in one unit cell, and β the index for the Cartesian coordinates. Compared to standard DFPT, which includes the linear responses of the external, Hartree, and electron–electron xc potential, we here include an additional contribution from the electron–photon exchange potential $v_{\mathrm{pxLDA}}\left(r\right)$. For the electron–photon interaction, the key piece to include in the standard DFPT is the response of the electron–photon exchange potential (within LDA) to the change of the electron density ρ(r),

$$\frac{\delta v_{\mathrm{pxLDA}}\left(r\right)}{\delta \rho (r^{\prime })}={\left(\frac{3}{8\pi }\right)}^{2/3}\sum_{\alpha =1}^{M_{p}}\frac{\pi {\tilde{\lambda }}_{\alpha }^{2}}{3{\tilde{\omega }}_{\alpha }^{2}}{\left[\rho (r^{\prime })\right]}^{-1/3}\left\{{\left({\tilde{\varepsilon }}_{\alpha }\cdot \nabla^{\prime }\right)}^{2}\frac{1}{|r-r^{\prime }|}\right\},$$

which can be represented in any basis set. In this work, we have included this response term in terms of the plane-wave basis set in the QE PHONON package (66, 67), in addition to the electron–photon exchange potential $v_{\mathrm{pxLDA}}\left(r\right)$, which we have also implemented in the QE PW package.

Upon solving the iterative and linear system, we obtain two terms resulting from the atomic displacement perturbation: 1) the change in the wave function $\partial \psi_{i}\left(r\right)/\partial \mu$ and 2) the change in the KS potential $\partial v_{\mathrm{KS}}\left(r\right)/\partial \mu$. The former can be used to compute the induced electron density from the atomic displacement, ∂ρ(r)/∂μ, which is then used to compute the second derivative of the total energy of the matter $E_{\mathrm{tot}}$ with respect to the atomic displacement, i.e., $\partial^{2}E_{\mathrm{tot}}/\partial \mu^{\prime }\partial \mu$ (68). These derivatives are then employed, given a phonon wave number q, to construct the dynamical matrix, whose eigenvalues are the square of the phonon frequencies $\omega_{\nu q}$, where ν is the index for phonon modes. The dynamical matrices computed on a coarse q-grid in the Brillouin zone also allow us to construct the interatomic force constants (68). Furthermore, the perturbation potential due to the atomic displacement $\partial v_{\mathrm{KS}}\left(r\right)/\partial \mu$ allows us to compute electron–phonon coupling strengths for specific electronic states and phonon modes (69).

Using the information for electrons and phonons, together with electron–phonon coupling strengths, we can compute the superconductivity of the material coupled to photon modes by solving the anisotropic Migdal-Eliashberg equations (70) based on first-principles methods (49, 54). This calculation enables us to obtain the momentum-resolved superconducting gaps $\Delta_{nk}$ and, consequently, determine the superconducting transition temperature T_c_ (49, 54). While solving the anisotropic Eliashberg equation can be computationally challenging, we can use the Wannier-interpolation method for electronic states and electron–phonon couplings implemented in open-source codes (71–74). The main quantities used in solving the anisotropic Eliashberg equations from first principles are as follows (please refer to refs. 54 and 72 for more details). The anisotropic Eliashberg equations (the mass renormalization function Z and the superconducting gap Δ) to solve are

$$\begin{array}{cc} Z(nk,i\omega_{l}) & =1+\frac{\pi T}{N_{F}\omega_{l}}\sum_{mk^{\prime },l^{\prime }}\frac{\omega_{l^{\prime }}}{\sqrt{\omega_{l^{\prime }}^{2}+\Delta^{2}\left(mk^{\prime },i\omega_{l^{\prime }}\right)}}\\ & \;\times \lambda \left(nk,mk^{\prime },l-l^{\prime }\right)\delta \left(\epsilon_{nk}\right),\\ Z\left(nk,i\omega_{l}\right)\Delta \left(nk,i\omega_{l}\right) & =\frac{\pi T}{N_{F}\omega_{l}}\sum_{mk^{\prime },l^{\prime }}\frac{\Delta (mk^{\prime },i\omega_{l^{\prime }})}{\sqrt{\omega_{l^{\prime }}^{2}+\Delta^{2}\left(mk^{\prime },i\omega_{l^{\prime }}\right)}}\\ & \;\times \left[\lambda \left(nk,mk^{\prime },l-l^{\prime }\right)-N_{F}V_{nk,mk^{\prime }}\right]\delta \left(\epsilon_{nk}\right), \end{array}$$

where $i\omega_{l}=i\left(2l+1\right)\pi T$ is the fermion Matsubara frequency (l is an integer), T the temperature, and $N_{F}$ the density of states at the Fermi level. $V_{nk,mk^{\prime }}$ is the static screened Coulomb interaction between the electronic states nk and $mk^{\prime }$, and here we use an effective Coulomb parameter $\mu^{*}$ to take this interaction into account. The anisotropic electron–phonon coupling matrix $\lambda (nk,mk^{\prime },l-l^{\prime })$ is defined as

$$\lambda \left(nk,mk^{\prime },l-l^{\prime }\right)=\int_{0}^{\infty }d\omega \frac{2\omega }{{\left(\omega_{l}-\omega_{l^{\prime }}\right)}^{2}+\omega^{2}}\alpha^{2}F\left(nk,mk^{\prime },\omega \right),$$

where the anisotropic Eliashberg electron–phonon spectral function $\alpha^{2}F\left(nk,mk^{\prime },\omega \right)$ can be computed using

$$\alpha^{2}F\left(nk,mk^{\prime },\omega \right)=N_{F}\sum_{\nu }{\left|g_{mn,\nu }^{\mathrm{SE}}\left(k,q\right)\right|}^{2}\delta \left(\omega -\omega_{\nu ,q=k-k^{\prime }}\right),$$

with the screend electron–phonon matrix element $g_{mn,\nu }^{\mathrm{SE}}\left(k,q\right)={\left(1/2\omega_{\nu q}\right)}^{1/2}g_{mn,\nu }\left(k,q\right)$. The associated isotropic Eliashberg spectral function $\alpha^{2}F\left(\omega \right)$ can be obtained by

$$\alpha^{2}F\left(\omega \right)=\sum_{nk,mk^{\prime }}W_{nk}W_{mk^{\prime }}\alpha^{2}F\left(nk,mk^{\prime },\omega \right),$$

where $W_{nk}=\delta \left(\epsilon_{nk}\right)/N_{F}$. The cumulative electron–phonon coupling strength is given by

$$\lambda \left(\omega \right)=2\int_{0}^{\omega }d\omega^{\prime }\frac{\alpha^{2}F\left(\omega^{\prime }\right)}{\omega^{\prime }},$$

from which the total electron–phonon coupling strength λ can be computed by setting ω beyond the highest phonon frequency of the material. The band- and wave-vector-dependent electron–phonon coupling strength $\lambda_{nk}$ for the electronic state |nk⟩ is defined as $\lambda_{nk}=\sum_{mk^{\prime }}W_{mk^{\prime }}\lambda \left(nk,mk^{\prime },l-l^{\prime }=0\right)$.

### Computational Details.

The ground state of MgB_2_ outside a cavity is obtained using the LDA (PZ) functional (75), together with the norm-conserving pseudopotential, in QE (66, 67). The DFT-relaxed crystal structure of MgB_2_ has a hexagonal lattice with the lattice constants a=3.0264 Å and c=3.4636 Å where the lattice constant a is parallel to the Boron plane and c is perpendicular to the plane. The atomic positions in crystal coordinates for the Magnesium and two Boron atoms in the unit cell are (0,0,0), (1/3,2/3,1/2), and (2/3,1/3,1/2), respectively. We use the kinetic energy cutoff of 60 Rydberg and the Monkhorst-Pack k-grid of 24×24×24 k-points centered at the Γ point to converge the total energy within the error of 10 meV per atom. Here, we use the Marzari–Vanderbilt smearing function (76) with a smearing value of 0.02 Rydberg. We calculate the skin depth using the Beer–Lambert law, δe(ω)=c/(ωImn(ω)), where n(ω) is the refractive of bulk MgB_2_. The refractive index is calculated as $n\left(\omega \right)=\sqrt{\epsilon_{M}\left(\omega \right)}$, where $\epsilon_{M}\left(\omega \right)$ is the macroscopic dielectric function of bulk MgB_2_ in the long-wavelength limit. This complex dielectric function is calculated using linear response in DFT within the RPA. The RPA is expected to work well because of the metallic nature of the MgB_2_ sample (77, 78). For the dielectric function calculation, we use a Monkhorst-Pack k-grid of 30×30×30k-points, a Gaussian broadening of 0.2 eV, and include 120 electronic bands. These dielectric function calculations were performed using the QE package. The phonon properties are computed on a coarse uniform q-grid of 6×6×6 q-point using DFPT implemented in the QE PHONON package (66, 67). The computed dynamical matrices on the coarse uniform q-grid are used to construct the interatomic force constants, which are then used to interpolate the phonon dispersion shown in the main text. The Wannier functions are constructed on a coarse k-grid of 6×6×6 k-points using Wannier90 package (71) with the initial projections, which are the $p_{z}$ orbital for each Boron atom and three s orbitals located at (0,1.0,0.5), (0.0,0.5,0.5), and (0.5,0.5,0.5) in crystal coordinates. The electron–phonon couplings in the Bloch basis are computed on the coarse k- and q-grids of 6×6×6, and then are used to construct the electron–phonon couplings in the Wannier basis, which are used as a small set to interpolate electron–phonon couplings at arbitrary k and q point; the electron–phonon couplings are calculated using EPW (72). The momentum-resolved superconducting gaps are obtained by solving the anisotropic Eliashberg equation implemented in EPW (54, 72). We use a fine k-grid of 60×60×60 points and a fine q-grid of 30×30×30. For solving the anisotropic Eliashberg equation, we use the following conditions: the effective Coulomb potential $\mu^{*}$ is 0.16, the Matsubara frequency cutoff is 1 eV, and the Dirac broadening for electrons is 0.1 eV, while that for phonons is 0.05 meV. For the ground states of MgB_2_ inside the cavity, we use the same conditions as those outside the cavity, while additionally including the electron–photon exchange potential within the LDA in solving the KS Hamiltonian. For the out-of-plane cavity with one effective photon mode, we use the photon frequency of 0.03675 Hartree (70 meV), and vary the (dimensionless) ratio of the mode strength and photon frequency, i.e., $\lambda_{\alpha }/\omega_{\alpha }$, from 0.14 to 1.4. Note that the results obtained from our method do not change if we use another photon frequency (e.g., 700 meV) but keep the same ratio of the mode strength and photon frequency. For the in-plane cavity with two effective photon modes, we use the same conditions, but its electron–photon exchange potential is weighted by 1/2.

## Data Availability

All study data are included in the main text.
